# Acute Subarachnoid Hemorrhage and Cardiac Abnormalities: Takotsubo Cardiomyopathy or Neurogenic Stunned Myocardium? a case report

**DOI:** 10.1186/1757-1626-3-81

**Published:** 2010-02-20

**Authors:** C Franco, B Khaled, L Afonso, M Raufi

**Affiliations:** 1 Department of Internal Medicine, Wayne State University School of Medicine, Detroit, Michigan,48201, USA; 2 Department of Cardiology. Wayne State University, Detroit Medical Center. Detroit, Michigan. 48201 USA

## Abstract

**Introduction:**

Cardiac abnormalities can be seen with subarachnoid hemorrhage. To date, there have been isolated case reports of transient left ventricular apical ballooning cardiomyopathy, also known as Takotsubo cardiomyopathy in patients suffering from subarachnoid hemorrhage.

**Case presentation:**

An adult female was brought to the emergency department with somnolence. A 3 × 3 mm ruptured basilar aneurysm was found and successfully embolized. Two days after the patient developed acute heart failure. Troponin-I was elevated to 4.2 (normal <0.4). On ECG, new symmetric T wave inversion in V3, V4, V5 with prolonged QT were evident. Transthoracic echocardiogram showed severe systolic dysfunction with an ejection fraction of 20% and akinetic apex along with the distal left ventricular segments, consistent with Takotsubo cardiomyopathy. Myocardial contrast echocardiography showed a decrease in capillary blood flow and volume in the akinetic areas with delayed contrast replenishment, sparing the basal segments. A repeat study 2 weeks later showed near normalization of the perfusion parameters. The patient improved with medical management. A repeat echocardiogram, a month later revealed an ejection fraction of 45% with no identifiable wall motion abnormality.

**Conclusion:**

Our case, as well as others reported previously, supports the diagnosis of Takotsubo cardiomyopathy in patients with Subarachnoid Hemorrhage who fulfill the clinical and imaging description of this syndrome.

## Introduction

Cardiac abnormalities can be seen with subarachnoid hemorrhage (SAH). ECG changes are seen in 50 to 100% of patients, and include deep T-wave inversion and QT-prolongation. Troponin elevation is seen in 20% to 40% and regional wall motion abnormalities (RWMA) in 10% of patients.

To date, there have been only 8 case reports of transient left ventricular apical ballooning cardiomyopathy (TLVABC), also known as Takotsubo cardiomyopathy in patients suffering from SAH. In contrast to the typically described neurogenic stunned myocardium where the basal and middle ventricular portions of the anteroseptal and anterior walls are involved, Takotsubo cardiomyopathy involves the apical and distal walls of the left ventricle.

## Case presentation

An adult female was brought to the emergency department with somnolence. Physical examination showed an adult female, with no focal neurological signs. Head CT revealed subarachnoid hemorrhage and an angiogram showed a 3 × 3 mm ruptured basilar aneurysm that was successfully embolized.

Two days after, the patient became dyspneic, was found hypoxemic, and had to be intubated. Physical examination and CXR were both consistent with acute heart failure. Troponin-I was 4.2 ng/ml (normal <0.4 ng/ml). On ECG, new symmetric T wave inversion in V3, V4, V5 with prolonged QT were evident. Transthoracic echocardiogram showed severe systolic dysfunction with an ejection fraction of 20% and akinetic apex along with the distal left ventricular segments, consistent with TLVABC. Myocardial contrast echocardiography showed a decrease in capillary blood flow and volume in the akinetic areas with delayed contrast replenishment, sparing the basal segments. Medical management was initiated (ACEI, Beta Blockers, Diuresis and Vasopressors). The patient improved, and eight days later was extubated. A repeat echo, a month later revealed an ejection fraction of 45% with no identifiable wall motion abnormality. The myocardial contrast echocardiography was repeated 2 weeks later and showed normalization of the perfusion parameters. The patient was discharged to a sub-acute care facility in stable condition.

## Discussion

In 2006, the American Heart Association defined Cardiomyopathies as a heterogeneous group of diseases of the myocardium associated with mechanical and/or electrical dysfunction that usually (but not invariably) exhibit inappropriate ventricular hypertrophy or dilatation and are due to a variety of causes that frequently are genetic. Cardiomyopathies either are confined to the heart or are a part of generalized systemic disorders, often leading to cardiovascular death or progressive heart failure-related disability. For research purposes, cardiomyopathies need muscle biopsy confirmation.

TLVABC (Takotsubo cardiomyopathy) is characterized by transient left ventricular dysfunction with complete regression upon follow-up among all survivors, in the absence of significant coronary artery disease. The exact mechanism is still unknown. Proposed hypotheses include multivessel coronary vasospasm, abnormalities in coronary microvascular function, and catecholamine-mediated cardiotoxicity. Typically, this condition is associated with sudden emotional or physical stress, accompanied by a catecholamine surge. This hypersympathetic state can be seen in other entities including Subarachnoid Hemorrhage (SAH). Although, a cardiac syndrome exemplary for TLVABC has been reported with SAH, to date, intracranial pathologies have been an exclusion criteria for the diagnosis of TLVABC.

Our case, as well as others reported previously, supports the diagnosis of TLVABC in patients with SAH who fulfill the clinical and imaging description of this syndrome. It also illustrates the varied presentations of cardiac dysfunction encountered in hypercatecholaminergic scenarios, argues for the standardization of terminology and calls for an all-inclusive definition of Takotsubo Syndrome that encompasses neurogenic stunned myocardium as well.

## Competing interests

The authors declare that they have no competing interests.

## Authors' contributions

Carlos Franco MD: writing of the case Khaled Bachour MD: writing of the case Luis Afonso MD: supervison and correction of the case Mohammed Raufi MD: patient was originally seen by him.

**Figure 1:**
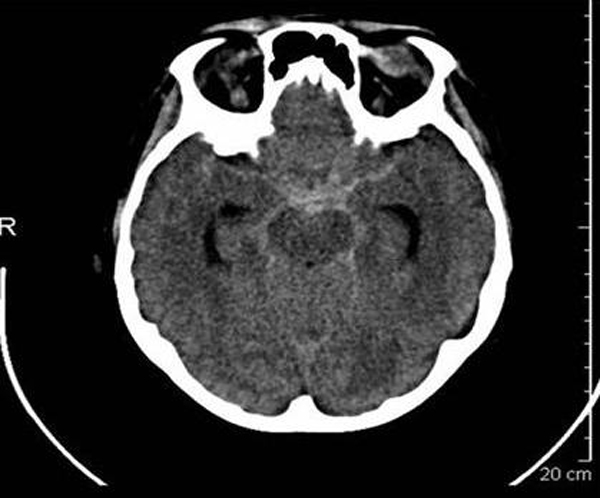
**Noncontrast CT showing subarachnoid hemorrhage**.

**Figure 2:**
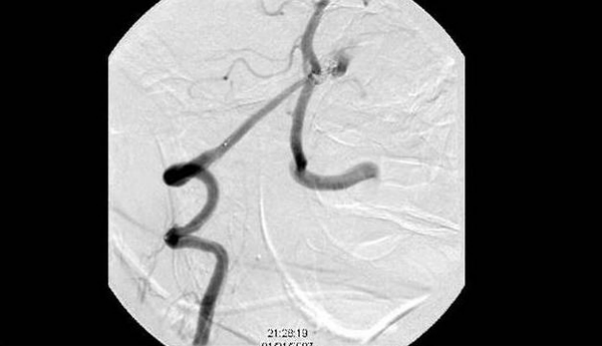
**Ruptured basilar fenestration aneurysm off of the right vertebral to basilar artery, 3 × 3 mm with a 2 mm neck pointing anterior and left**.

## Consent

Written informed consent could not be obtained. All reasonable attempts to obtain consent from the patient were made but the patient was a foreign national who after discharge returned to her home country. Every effort to preserve the patient's anonymity was made. There is no reason to believe that the patient would object to publication.
